# Supplementary material for: From data to decisions: Predicting inpatient burn mortality with advanced classification models
Source: PLoS One. 2026 Jan 2;21(1):e0338564. doi: 10.1371/journal.pone.0338564 (PMC12758681; doi:10.1371/journal.pone.0338564)
Supplement: S1 Table — Detailed descriptive statistics and characteristics of all variables used in the study. (DOCX) [file pone.0338564.s001.docx]

## **S1 Table. Features Used for Model Development.**

The following table presents the 94 features selected after data preprocessing, including the most influential variables identified through feature weighting techniques. These features were extracted from the burn registry data of the Injuries and Burn Subspecialized Teaching Hospital, affiliated with Isfahan University of Medical Sciences and were used to construct the predictive models.

| **Feature** | **Group** | **Name** | **Type** | **Value** | **Freq** | **%** |
| --- | --- | --- | --- | --- | --- | --- |
| F1 | **Demographic** | Age (years) | Continuous | Mean ± SD: 33.2 ± 19.17 |  |  |
|  |  |  | Categorical (ordinal) | 1=Child(<=12) | 114 | 17.22 |
|  |  |  |  | 2=Adolescent(12<years<=18) | 45 | 6.80 |
|  |  |  |  | 3= Young Adult(18<years<=35) | 185 | 27.95 |
|  |  |  |  | 4= Middle-aged(35<years<=60) | 270 | 40.79 |
|  |  |  |  | 5=Elderly(>60) | 48 | 7.25 |
|  |  |  |  | MISS | 0 | 0.00 |
| **F2** |  | Gender | Categorical (binary) | 1=Male | 482 | 72.81 |
|  |  |  |  | 2=Female | 180 | 27.19 |
|  |  |  |  | MISS | 0 | 0.00 |
| **F3** |  | Ethnicity | Categorical (nominal) | 1=Persian | 323 | 48.79 |
|  |  |  |  | 2=Afghan | 14 | 2.11 |
|  |  |  |  | 3=Turk | 55 | 8.31 |
|  |  |  |  | 4=Kurd | 38 | 5.74 |
|  |  |  |  | 5=Arab | 36 | 5.44 |
|  |  |  |  | 6=Lur | 193 | 29.15 |
|  |  |  |  | 7=Turkmen | 1 | 0.15 |
|  |  |  |  | 8=Other | 2 | 0.30 |
|  |  |  |  | MISS | 0 | 0.00 |
| **F4** |  | Education Level | Categorical (ordinal) | 1=Preschool Child | 71 | 10.73 |
|  |  |  |  | 2=Illiterate | 61 | 9.21 |
|  |  |  |  | 3=Literacy Program Participant | 1 | 0.15 |
|  |  |  |  | 4=Elementary School | 147 | 22.21 |
|  |  |  |  | 5=Middle School | 139 | 21.00 |
|  |  |  |  | 6=High School Diploma | 151 | 22.81 |
|  |  |  |  | 7=Associate's Degree | 25 | 3.78 |
|  |  |  |  | 8=Bachelor's Degree | 54 | 8.16 |
|  |  |  |  | 9=Master's Degree | 8 | 1.21 |
|  |  |  |  | 10=Doctorate | 5 | 0.76 |
|  |  |  |  | MISS | 0 | 0.00 |
| **F5** |  | Marital Status | Categorical (nominal) | 1=Single | 261 | 39.43 |
|  |  |  |  | 2=Married | 361 | 54.53 |
|  |  |  |  | 3=Divorced | 22 | 3.32 |
|  |  |  |  | 4=Widowed | 18 | 2.72 |
|  |  |  |  | MISS | 0 | 0.00 |
| **F6** |  | Under 18 Years Old? | Categorical (binary) | 1=Yes | 141 | 21.30 |
|  |  |  |  | 2=No | 517 | 78.10 |
|  |  |  |  | MISS | 4 | 0.60 |
| **F7** |  | Under 6 Years Old? | Categorical (binary) | 1=Yes | 80 | 12.08 |
|  |  |  |  | 2=No | 562 | 84.89 |
|  |  |  |  | MISS | 20 | 3.02 |
| **F8** |  | Height | Continuous | Mean ± SD: 160.01 ± 27.98 |  |  |
|  |  |  | Categorical (ordinal) | 1=Short(<160) | 155 | 23.41 |
|  |  |  |  | 2=Average(160<=H<=180) | 438 | 66.16 |
|  |  |  |  | 3=Tall(>180) | 57 | 8.61 |
|  |  |  |  | MISS | 12 | 1.81 |
| **F9** |  | Weight | Continuous | Mean ± SD: 65.03 ± 25.42 |  |  |
|  |  |  | Categorical (ordinal) | 1=Underweight(<50) | 131 | 19.79 |
|  |  |  |  | 2=Normal weight(50<=W<70) | 164 | 24.77 |
|  |  |  |  | 3=Overweight(70<=W<=90) | 300 | 45.32 |
|  |  |  |  | 4=Obese(>90) | 62 | 9.37 |
|  |  |  |  | MISS | 5 | 0.76 |
| **F10** |  | BMI (years > 15 ) | Continuous | Mean ± SD: 25.13 ± 4.57 MISS | 544 118 | 82.18 17.82 |
|  |  |  | Categorical (ordinal) | 1=Under 15 years | 84 | 12.69 |
|  |  |  |  | 2=Underweight(<18.5) | 31 | 4.68 |
|  |  |  |  | 3=Normal weight(18.5<=BMI<25) | 255 | 38.52 |
|  |  |  |  | 4=Overweight(25<=BMI<30) | 176 | 26.59 |
|  |  |  |  | 5=Obese(>30) | 82 | 12.39 |
|  |  |  |  | MISS | 34 | 5.14 |
| **F11** |  | Z-Score (years < 15) | Discrete | 1=-3 | 3 | 0.45 |
|  |  |  |  | 2=-2 | 15 | 2.27 |
|  |  |  |  | 3=-1 | 21 | 3.17 |
|  |  |  |  | 4=0 | 3 | 0.45 |
|  |  |  |  | 5=1 | 40 | 6.04 |
|  |  |  |  | 6=2 | 19 | 2.87 |
|  |  |  |  | 7=3 | 11 | 1.66 |
|  |  |  |  | 8=Over 15 years | 516 | 77.95 |
|  |  |  |  | MISS | 34 | 5.14 |
| **F12** |  | Health Insurance Status | Categorical (nominal) | 1=NO | 78 | 11.78 |
|  |  |  |  | 2=YES(Basic Insurance) | 444 | 67.07 |
|  |  |  |  | 3=Basic and Supplemental | 137 | 20.69 |
|  |  |  |  | MISS | 3 | 0.45 |
| **F13** | **Medical History** | Vision Limitation | Categorical (binary) | 1=NO | 504 | 76.13 |
|  |  |  |  | 2=YES | 158 | 23.87 |
|  |  |  |  | MISS | 0 | 0.00 |
| **F14** |  | Limb Amputation | Categorical (binary) | 1=NO | 603 | 91.09 |
|  |  |  |  | 2=YES | 58 | 8.76 |
|  |  |  |  | MISS | 1 | 0.15 |
| **F15** |  | Allergy Status | Categorical (binary) | 1=NO | 506 | 76.44 |
|  |  |  |  | 2=YES | 155 | 23.41 |
|  |  |  |  | MISS | 1 | 0.15 |
| **F16** |  | Cardiovascular Disease | Categorical (binary) | 1=NO | 87 | 13.14 |
|  |  |  |  | 2=YES | 15 | 2.27 |
|  |  |  |  | MISS | 560 | 84.59 |
| **F17** |  | Thyroid Disease | Categorical (binary) | 1=NO | 84 | 12.69 |
|  |  |  |  | 2=YES | 18 | 2.72 |
|  |  |  |  | MISS | 560 | 84.59 |
| **F18** |  | Diabetes | Categorical (binary) | 1=NO | 88 | 13.29 |
|  |  |  |  | 2=YES | 14 | 2.11 |
|  |  |  |  | MISS | 560 | 84.59 |
| **F19** |  | Respiratory Disease | Categorical (binary) | 1=NO | 92 | 13.90 |
|  |  |  |  | 2=YES | 10 | 1.51 |
|  |  |  |  | MISS | 560 | 84.59 |
| **F20** |  | Neurological Disease | Categorical (binary) | 1=NO | 90 | 13.60 |
|  |  |  |  | 2=YES | 12 | 1.81 |
|  |  |  |  | MISS | 560 | 84.59 |
| **F21** |  | Hyperlipidemia | Categorical (binary) | 1=NO | 96 | 14.50 |
|  |  |  |  | 2=YES | 6 | 0.91 |
|  |  |  |  | MISS | 560 | 84.59 |
| **F22** |  | Renal Disease | Categorical (binary) | 1=NO | 90 | 13.60 |
|  |  |  |  | 2=YES | 12 | 1.81 |
|  |  |  |  | MISS | 560 | 84.59 |
| **F23** |  | Psychiatric Disease | Categorical (binary) | 1=NO | 81 | 12.24 |
|  |  |  |  | 2=YES | 21 | 3.17 |
|  |  |  |  | MISS | 560 | 84.59 |
| **F24** |  | Gastrointestinal Disease | Categorical (binary) | 1=NO | 88 | 13.29 |
|  |  |  |  | 2=YES | 14 | 2.11 |
|  |  |  |  | MISS | 560 | 84.59 |
| **F25** |  | COVID-19 Infection | Categorical (nominal) | 1=NO | 378 | 57.10 |
|  |  |  |  | 2=YES | 264 | 39.88 |
|  |  |  |  | 3=Unknown | 17 | 2.57 |
|  |  |  |  | MISS | 3 | 0.45 |
| **F26** |  | Alcohol Consumption History | Categorical (nominal) | 1=NO | 585 | 88.37 |
|  |  |  |  | 2=YES | 74 | 11.18 |
|  |  |  |  | 3=Unknown | 1 | 0.15 |
|  |  |  |  | MISS | 2 | 0.30 |
| **F27** |  | Smoking History | Categorical (nominal) | 1=NO | 399 | 60.27 |
|  |  |  |  | 2=Cigarette | 185 | 27.95 |
|  |  |  |  | 3=Hookah | 50 | 7.55 |
|  |  |  |  | 4=Cigarette and Hookah | 25 | 3.78 |
|  |  |  |  | MISS | 3 | 0.45 |
| **F28** |  | Smoking History: Daily Cigarette Consumption | Categorical (nominal) | 1=No (Does not apply) | 449 | 67.82 |
|  |  |  |  | 2=1 to 10 cigarettes | 62 | 9.37 |
|  |  |  |  | 3=11 to 20 cigarettes | 121 | 18.28 |
|  |  |  |  | 4=More than one pack | 22 | 3.32 |
|  |  |  |  | MISS | 8 | 1.21 |
| **F29** |  | Drug Use History | Categorical (binary) | 1=NO | 532 | 80.36 |
|  |  |  |  | 2=YES | 122 | 18.43 |
|  |  |  |  | MISS | 8 | 1.21 |
| **F30** | **Admission and Referral Data** | Prior Burn-Related Hospitalization | Categorical (binary) | 1=NO | 186 | 28.10 |
|  |  |  |  | 2=YES | 474 | 71.60 |
|  |  |  |  | MISS | 2 | 0.30 |
| **F31** |  | Transport Mode | Categorical (nominal) | 1=Referring Hospital Ambulance | 238 | 35.95 |
|  |  |  |  | 2=115 Emergency Service Ambulance | 97 | 14.65 |
|  |  |  |  | 3=Private Ambulance | 126 | 19.03 |
|  |  |  |  | 4=Air Ambulance | 3 | 0.45 |
|  |  |  |  | 5=Taxi | 6 | 0.91 |
|  |  |  |  | 6=Personal Vehicle | 183 | 27.64 |
|  |  |  |  | 7=On Foot | 1 | 0.15 |
|  |  |  |  | MISS | 8 | 1.21 |
| **F32** |  | Braden Score | Continuous | Mean ± SD: 15.27 ± 2.90 |  |  |
|  |  |  | Categorical (ordinal) | 1=Low Risk(<=12) | 147 | 22.21 |
|  |  |  |  | 2=Moderate Risk(12<B<18) | 397 | 59.97 |
|  |  |  |  | 3=High Risk(>=18) | 113 | 17.07 |
|  |  |  |  | MISS | 5 | 0.76 |
| **F33** |  | Morse (Humpty Dumpty) Score | Continuous | Mean ± SD: 27.44 ± 12.21 |  |  |
|  |  |  | Categorical (ordinal) | 1=Low Risk(<=24) | 313 | 47.28 |
|  |  |  |  | 2=Moderate Risk(24<M<=44) | 274 | 41.39 |
|  |  |  |  | 3=High Risk(>44) | 70 | 10.57 |
|  |  |  |  | MISS | 5 | 0.76 |
| **F34** |  | Wells Score | Continuous | Mean ± SD: 3.24 ± 1.41 |  |  |
|  |  |  | Categorical (ordinal) | 1=Low Risk(We=0) | 0 | 0.00 |
|  |  |  |  | 2=Moderate Risk(0<We<=2) | 230 | 34.74 |
|  |  |  |  | 3=High Risk(>2) | 422 | 63.75 |
|  |  |  |  | MISS | 10 | 1.51 |
| **F35** |  | Tetanus Toxoid Injection | Categorical (binary) | 1=NO | 316 | 47.73 |
|  |  |  |  | 2=YES | 341 | 51.51 |
|  |  |  |  | MISS | 5 | 0.76 |
| **F36** |  | Tetanus Immunoglobulin Injection | Categorical (binary) | 1=NO | 314 | 47.43 |
|  |  |  |  | 2=YES | 343 | 51.81 |
|  |  |  |  | MISS | 5 | 0.76 |
| **F37** | **Pre-hospital Care** | Pre-hospital Medical Care | Categorical (nominal) | 1=NO | 188 | 28.40 |
|  |  |  |  | 2=YES | 469 | 70.85 |
|  |  |  |  | 3=Unknown | 3 | 0.45 |
|  |  |  |  | MISS | 2 | 0.30 |
| **F38** |  | IV Fluid Therapy | Categorical (binary) | 1=NO | 242 | 36.56 |
|  |  |  |  | 2=YES | 420 | 63.44 |
|  |  |  |  | MISS | 0 | 0.00 |
| **F39** |  | Wound Dressing | Categorical (binary) | 1=NO | 262 | 39.58 |
|  |  |  |  | 2=YES | 400 | 60.42 |
|  |  |  |  | MISS | 0 | 0.00 |
| **F40** |  | Vital Signs Monitoring | Categorical (binary) | 1=NO | 269 | 40.63 |
|  |  |  |  | 2=YES | 393 | 59.37 |
|  |  |  |  | MISS | 0 | 0.00 |
| **F41** |  | Urinary Catheter | Categorical (binary) | 1=NO | 391 | 59.06 |
|  |  |  |  | 2=YES | 271 | 40.94 |
|  |  |  |  | MISS | 0 | 0.00 |
| **F42** |  | Pain Management | Categorical (binary) | 1=NO | 371 | 56.04 |
|  |  |  |  | 2=YES | 291 | 43.96 |
|  |  |  |  | MISS | 0 | 0.00 |
| **F43** |  | Nasogastric Tube | Categorical (binary) | 1=NO | 570 | 86.10 |
|  |  |  |  | 2=YES | 92 | 13.90 |
|  |  |  |  | MISS | 0 | 0.00 |
| **F44** |  | Wound Debridement | Categorical (binary) | 1=NO | 619 | 93.50 |
|  |  |  |  | 2=YES | 43 | 6.50 |
|  |  |  |  | MISS | 0 | 0.00 |
| **F45** |  | Antibiotic Therapy | Categorical (binary) | 1=NO | 594 | 89.73 |
|  |  |  |  | 2=YES | 68 | 10.27 |
|  |  |  |  | MISS | 0 | 0.00 |
| **F46** |  | Intubation | Categorical (binary) | 1=NO | 595 | 89.88 |
|  |  |  |  | 2=YES | 67 | 10.12 |
|  |  |  |  | MISS | 0 | 0.00 |
| **F47** | **Burn Care in ER** | Initial Attending Physician | Categorical (nominal) | 1=General Practitioner | 561 | 84.74 |
|  |  |  |  | 2=Emergency Medicine Specialist | 89 | 13.44 |
|  |  |  |  | 3=Infectious Disease Specialist | 1 | 0.15 |
|  |  |  |  | MISS | 11 | 1.66 |
| **F48** |  | Tetanus Prophylaxis | Categorical (binary) | 1=NO | 331 | 50.00 |
|  |  |  |  | 2=YES | 316 | 47.73 |
|  |  |  |  | MISS | 15 | 2.27 |
| **F49** |  | Wound Dressing in ER | Categorical (binary) | 1=NO | 18 | 2.72 |
|  |  |  |  | 2=YES | 629 | 95.02 |
|  |  |  |  | MISS | 15 | 2.27 |
| **F50** |  | Blood Culture Test | Categorical (binary) | 1=NO | 494 | 74.62 |
|  |  |  |  | 2=YES | 153 | 23.11 |
|  |  |  |  | MISS | 15 | 2.27 |
| **F51** |  | Wound Culture Test | Categorical (binary) | 1=NO | 486 | 73.41 |
|  |  |  |  | 2=YES | 161 | 24.32 |
|  |  |  |  | MISS | 15 | 2.27 |
| **F52** |  | Urinary Catheterization | Categorical (binary) | 1=NO | 506 | 76.44 |
|  |  |  |  | 2=YES | 141 | 21.30 |
|  |  |  |  | MISS | 15 | 2.27 |
| **F53** |  | Medication Therapy | Categorical (binary) | 1=NO | 437 | 66.01 |
|  |  |  |  | 2=YES | 210 | 31.72 |
|  |  |  |  | MISS | 15 | 2.27 |
| **F54** |  | ECG | Categorical (binary) | 1=NO | 440 | 66.47 |
|  |  |  |  | 2=YES | 207 | 31.27 |
|  |  |  |  | MISS | 15 | 2.27 |
| **F55** |  | Nasogastric Tube Placement | Categorical (binary) | 1=NO | 559 | 84.44 |
|  |  |  |  | 2=YES | 88 | 13.29 |
|  |  |  |  | MISS | 15 | 2.27 |
| **F56** |  | Injectable Pain Relief | Categorical (binary) | 1=NO | 579 | 87.46 |
|  |  |  |  | 2=YES | 68 | 10.27 |
|  |  |  |  | MISS | 15 | 2.27 |
| **F57** |  | Eye Consultation | Categorical (binary) | 1=NO | 529 | 79.91 |
|  |  |  |  | 2=YES | 128 | 19.34 |
|  |  |  |  | MISS | 5 | 0.76 |
| **F58** | **Post-ER** | Patient Status | Categorical (binary) | 1=Transferred to ICU | 116 | 17.52 |
|  |  |  |  | 2=Transferred to Inpatient Ward | 537 | 81.12 |
|  |  |  |  | MISS | 9 | 1.36 |
| **F59** | **Burn-Related Information** | Incident Location | Categorical (binary) | 1=Open Space | 239 | 36.10 |
|  |  |  |  | 2=Enclosed Space | 420 | 63.44 |
|  |  |  |  | MISS | 3 | 0.45 |
| **F60** |  | Burn Type | Categorical (nominal) | 1=Electrical | 33 | 4.98 |
|  |  |  |  | 2=Chemical | 8 | 1.21 |
|  |  |  |  | 3=Thermal Burn from Hot Liquids | 129 | 19.49 |
|  |  |  |  | 4=Thermal Burn from Hot Objects | 4 | 0.60 |
|  |  |  |  | 5=Thermal Burn from Explosion | 19 | 2.87 |
|  |  |  |  | 6=Thermal Burn from Flame | 30 | 4.53 |
|  |  |  |  | MISS | 439 | 66.31 |
| **F61** |  | Burn Type: Type of Hot Liquid | Categorical (nominal) | 1= Not Hot Liquids | 94 | 14.20 |
|  |  |  |  | 2=Boiling Water | 81 | 12.24 |
|  |  |  |  | 3=Broth | 14 | 2.11 |
|  |  |  |  | 4=Paint and Resin | 1 | 0.15 |
|  |  |  |  | 5=Radiator Water | 3 | 0.45 |
|  |  |  |  | 6=Rice Water | 4 | 0.60 |
|  |  |  |  | 7=Tea | 7 | 1.06 |
|  |  |  |  | 8=Hot Oil | 2 | 0.30 |
|  |  |  |  | 9=Milk | 4 | 0.60 |
|  |  |  |  | 10=Food | 7 | 1.06 |
|  |  |  |  | 11=Molten Material | 3 | 0.45 |
|  |  |  |  | MISS | 442 | 66.77 |
| **F62** |  | Burn Degree | Categorical (ordinal) | 1=First Degree | 4 | 0.60 |
|  |  |  |  | 2=Second Degree | 116 | 17.52 |
|  |  |  |  | 3=Third Degree | 146 | 22.05 |
|  |  |  |  | 4=Combination of Second and Third | 367 | 55.44 |
|  |  |  |  | 5=Combination of Second, Third, and Fourth | 4 | 0.60 |
|  |  |  |  | 6=Combination of Third and Fourth | 14 | 2.11 |
|  |  |  |  | MISS | 11 | 1.66 |
| **F63** |  | Total Burn Surface Area (TBSA) | Continuous | Mean ± SD: 38.17 ± 24.99 |  |  |
|  |  |  | Categorical (ordinal) | 1=Mild(<10) | 35 | 5.29 |
|  |  |  |  | 2=Moderate(10<=TBSA<30) | 247 | 37.31 |
|  |  |  |  | 3=Severe(30<=TBSA<50) | 174 | 26.28 |
|  |  |  |  | 4=Very Severe(>=50) | 205 | 30.97 |
|  |  |  |  | MISS | 1 | 0.15 |
| **F64** |  | Second-Degree Burns(SDB) | Continuous | Mean ± SD: 22.02 ± 13.36 |  |  |
|  |  |  | Categorical (ordinal) | 1=Mild(<10) | 42 | 6.34 |
|  |  |  |  | 2=Moderate(10<=SDB<30) | 294 | 44.41 |
|  |  |  |  | 3=Severe(30<=SDB<50) | 126 | 19.03 |
|  |  |  |  | 4=Very Severe(>=50) | 27 | 4.08 |
|  |  |  |  | 5=Zero (Other Degrees) | 167 | 25.23 |
|  |  |  |  | MISS | 6 | 0.91 |
| **F65** |  | Third-Degree Burns(TDB) | Continuous | Mean ± SD: 25.92 ± 24.81 |  |  |
|  |  |  | Categorical (ordinal) | 1=Mild(<10) | 143 | 21.60 |
|  |  |  |  | 2=Moderate(10<=TDB<30) | 207 | 31.27 |
|  |  |  |  | 3=Severe(30<=TDB<50) | 82 | 12.39 |
|  |  |  |  | 4=Very Severe(>=50) | 99 | 14.95 |
|  |  |  |  | 5=Zero (Other Degrees) | 125 | 18.88 |
|  |  |  |  | MISS | 6 | 0.91 |
| **F66** |  | Fourth-Degree Burns(FDB) | Continuous | Mean ± SD: 18.61 ± 12.44 |  |  |
|  |  |  | Categorical (ordinal) | 1=Mild(<10) | 3 | 0.45 |
|  |  |  |  | 2=Moderate(10<=FDB<30) | 11 | 1.66 |
|  |  |  |  | 3=Severe(30<=FDB<50) | 3 | 0.45 |
|  |  |  |  | 4=Very Severe(>=50) | 1 | 0.15 |
|  |  |  |  | 5=Zero (Other Degrees) | 635 | 95.92 |
|  |  |  |  | MISS | 9 | 1.36 |
| **F67** | **Burn Area** | Genitals | Categorical (binary) | 1=NO | 553 | 83.53 |
|  |  |  |  | 2=YES | 58 | 8.76 |
|  |  |  |  | MISS | 52 | 7.85 |
| **F68** |  | Head, Face, Neck | Categorical (binary) | 1=NO | 182 | 27.49 |
|  |  |  |  | 2=YES | 435 | 65.71 |
|  |  |  |  | MISS | 45 | 6.80 |
| **F69** |  | Hands | Categorical (binary) | 1=NO | 41 | 6.19 |
|  |  |  |  | 2=YES | 580 | 87.61 |
|  |  |  |  | MISS | 41 | 6.19 |
| **F70** |  | Feet | Categorical (binary) | 1=NO | 152 | 22.96 |
|  |  |  |  | 2=YES | 478 | 72.21 |
|  |  |  |  | MISS | 32 | 4.83 |
| **F71** |  | Anterior Torso | Categorical (binary) | 1=NO | 280 | 42.30 |
|  |  |  |  | 2=YES | 332 | 50.15 |
|  |  |  |  | MISS | 51 | 7.70 |
| **F72** |  | Posterior Torso | Categorical (binary) | 1=NO | 282 | 42.60 |
|  |  |  |  | 2=YES | 335 | 50.60 |
|  |  |  |  | MISS | 45 | 6.80 |
| **F73** | **Inhalation Injury** | Inhalation Injury | Categorical (binary) | 1=NO | 207 | 31.27 |
|  |  |  |  | 2=YES | 454 | 68.58 |
|  |  |  |  | MISS | 1 | 0.15 |
| **F74** | **cause of Inhalation Injury** | Third-Degree Burn of the Neck and Anterior Torso | Categorical (nominal) | 1=NO | 57 | 8.61 |
|  |  |  |  | 2=YES | 101 | 15.26 |
|  |  |  |  | 3=No Inhalation Injury | 207 | 31.27 |
|  |  |  |  | MISS | 297 | 44.86 |
| **F75** |  | Face Burn | Categorical (nominal) | 1=NO | 13 | 1.96 |
|  |  |  |  | 2=YES | 145 | 21.90 |
|  |  |  |  | 3=No Inhalation Injury | 207 | 31.27 |
|  |  |  |  | MISS | 297 | 44.86 |
| **F76** |  | Heat Inhalation | Categorical (nominal) | 1=NO | 52 | 7.85 |
|  |  |  |  | 2=YES | 106 | 16.01 |
|  |  |  |  | 3=No Inhalation Injury | 207 | 31.27 |
|  |  |  |  | MISS | 297 | 44.86 |
| **F77** | **Symptom of Inhalation Injury** | Cough | Categorical (nominal) | 1=NO | 249 | 37.61 |
|  |  |  |  | 2=YES | 44 | 6.65 |
|  |  |  |  | 3=No Inhalation Injury | 207 | 31.27 |
|  |  |  |  | MISS | 162 | 24.47 |
| **F78** |  | Shortness of Breath | Categorical (nominal) | 1=NO | 259 | 39.12 |
|  |  |  |  | 2=YES | 34 | 5.14 |
|  |  |  |  | 3=No Inhalation Injury | 207 | 31.27 |
|  |  |  |  | MISS | 162 | 24.47 |
| **F79** |  | Burnt Hair | Categorical (nominal) | 1=NO | 27 | 4.08 |
|  |  |  |  | 2=YES | 266 | 40.18 |
|  |  |  |  | 3=No Inhalation Injury | 207 | 31.27 |
|  |  |  |  | MISS | 162 | 24.47 |
| **F80** |  | Hoarseness | Categorical (nominal) | 1=NO | 155 | 23.41 |
|  |  |  |  | 2=YES | 138 | 20.85 |
|  |  |  |  | 3=No Inhalation Injury | 207 | 31.27 |
|  |  |  |  | MISS | 162 | 24.47 |
| **F81** |  | Low Oxygen Saturation | Categorical (nominal) | 1=NO | 195 | 29.46 |
|  |  |  |  | 2=YES | 98 | 14.80 |
|  |  |  |  | 3=No Inhalation Injury | 207 | 31.27 |
|  |  |  |  | MISS | 162 | 24.47 |
| **F82** | **Paraclinical Indicators** | Hemoglobin | Continuous | Mean ± SD: 15.51 ± 2.95 |  |  |
|  |  |  | Categorical (ordinal) | 1=Low(Men<13,Women<12) | 106 | 16.01 |
|  |  |  |  | 2=Normal(13<=Men<=17,12<=Women<=15) | 316 | 47.73 |
|  |  |  |  | 3=High(Men>17,Women>15) | 235 | 35.50 |
|  |  |  |  | MISS | 5 | 0.76 |
| **F83** |  | HCT | Continuous | Mean ± SD: 45.41 ± 7.21 |  |  |
|  |  |  | Categorical (ordinal) | 1=Low(Men<39,Women<35) | 92 | 13.90 |
|  |  |  |  | 2=Normal(39<=Men<=50,35<=Women<=45) | 366 | 55.29 |
|  |  |  |  | 3=High(Men>50,Women>45) | 199 | 30.06 |
|  |  |  |  | MISS | 5 | 0.76 |
| **F84** |  | WBC | Continuous | Mean ± SD: 15050.23 ± 8007.18 |  |  |
|  |  |  | Categorical (ordinal) | 1=Low(<4000) | 13 | 1.96 |
|  |  |  |  | 2=Normal(4000<=WBC<=11000) | 201 | 30.36 |
|  |  |  |  | 3=High(>11000) | 443 | 66.92 |
|  |  |  |  | MISS | 5 | 0.76 |
| **F85** |  | Platelets | Continuous | Mean ± SD: 281152.2 ± 161653.8 |  |  |
|  |  |  | Categorical (ordinal) | 1=Low(<150000) | 62 | 9.37 |
|  |  |  |  | 2=Normal(150000<=Pla<=450000) | 533 | 80.51 |
|  |  |  |  | 3=High(>450000) | 62 | 9.37 |
|  |  |  |  | MISS | 5 | 0.76 |
| **F86** |  | Sodium | Continuous | Mean ± SD: 138.7 ± 4.16 |  |  |
|  |  |  | Categorical (ordinal) | 1=Low(<135) | 95 | 14.35 |
|  |  |  |  | 2=Normal(135<=Sod<=145) | 527 | 79.61 |
|  |  |  |  | 3=High(>145) | 21 | 3.17 |
|  |  |  |  | MISS | 19 | 2.87 |
| **F87** |  | Potassium | Continuous | Mean ± SD: 4.21 ± 0.51 |  |  |
|  |  |  | Categorical (ordinal) | 1=Low(<3.5) | 28 | 4.23 |
|  |  |  |  | 2=Normal(3.5<=Pot<=5) | 574 | 86.71 |
|  |  |  |  | 3=High(>5) | 40 | 6.04 |
|  |  |  |  | MISS | 20 | 3.02 |
| **F88** |  | FBS | Continuous | Mean ± SD: 132.48 ± 51.83 |  |  |
|  |  |  | Categorical (ordinal) | 1=Normal(<100) | 164 | 24.77 |
|  |  |  |  | 2=Pre-Diabetes(100<=FBS<=125) | 193 | 29.15 |
|  |  |  |  | 3=Diabetes(>125) | 287 | 43.35 |
|  |  |  |  | MISS | 18 | 2.72 |
| **F89** |  | Creatinine | Continuous | Mean ± SD: 0.99 ± 0.33 |  |  |
|  |  |  | Categorical (ordinal) | 1=Low(Men<0.7,Women<0.6) | 311 | 46.98 |
|  |  |  |  | 2=Normal(0.7<=Men<=1.3,0.6<=Women<=1.1) | 266 | 40.18 |
|  |  |  |  | 3=High(Men>1.3,Women>1.1) | 68 | 10.27 |
|  |  |  |  | MISS | 17 | 2.57 |
| **F90** |  | BUN | Continuous | Mean ± SD: 15.41 ± 7.61 |  |  |
|  |  |  | Categorical (ordinal) | 1=Low(<7) | 9 | 1.36 |
|  |  |  |  | 2=Normal(7<=BUN<=20) | 533 | 80.51 |
|  |  |  |  | 3=High(>20) | 103 | 15.56 |
|  |  |  |  | MISS | 17 | 2.57 |
| **F91** |  | Albumin | Continuous | Mean ± SD: 3.71 ± 0.73 |  |  |
|  |  |  | Categorical (ordinal) | 1=Low(<3.5) | 217 | 32.78 |
|  |  |  |  | 2=Normal(3.5<=Alb<=5) | 406 | 61.33 |
|  |  |  |  | 3=High(>5) | 7 | 1.06 |
|  |  |  |  | MISS | 32 | 4.83 |
| **F92** |  | ESR | Continuous | Mean ± SD: 15.53 ± 22.42 |  |  |
|  |  |  | Categorical (ordinal) | 1=Normal(Men<=20,Women<=30) | 528 | 79.76 |
|  |  |  |  | 2=High(Men>20,Women>30) | 121 | 18.28 |
|  |  |  |  | MISS | 13 | 1.96 |
| **F93** | **Abbreviated Burn Severity Index(ABSI)** | Score | Discrete | Mean ± SD: 7.98 ± 3.05 |  | |
|  |  | Qualitative Classification | Categorical (ordinal) | 1=Very Low(<=3) | 22 | 3.32 |
|  |  |  |  | 2=Low(3< ABSI <=5) | 125 | 18.88 |
|  |  |  |  | 3=Moderate(5< ABSI <=7) | 184 | 27.79 |
|  |  |  |  | 4=Moderate to High(7< ABSI <=9) | 147 | 22.21 |
|  |  |  |  | 5=Severe(9< ABSI <=12) | 79 | 11.93 |
|  |  |  |  | 6=Very Severe(>12) | 104 | 15.71 |
|  |  |  |  | MISS | 1 | 0.15 |
| **F94** | **Patient Outcome** | Patient Outcome | Categorical (binary) | 1=DIED | 144 | 21.75 |
|  |  |  |  | 2=ALIVE | 507 | 76.59 |
|  |  |  |  | MISS | 11 | 1.66 |
| **Note:**   - The descriptive statistics presented in this table are based on the total initial cohort (n=662). The final modeling analysis was performed on a subset of 651 patients after excluding 11 individuals with missing outcome data. - This appendix provides essential context for understanding the input variables utilized during model training and evaluation. For further details about feature selection and preprocessing steps, please refer to the "Methods" section of this study. | | | | | | |
