# Supplementary material for: From data to decisions: Predicting inpatient burn mortality with advanced classification models
Source: PLoS One. 2026 Jan 2;21(1):e0338564. doi: 10.1371/journal.pone.0338564 (PMC12758681; doi:10.1371/journal.pone.0338564)
Supplement: S2 Table — Details of the grid search and selected parameters for each model. (DOCX) [file pone.0338564.s002.docx]

## **S2 Table. Hyperparameter Tuning Search Space and Optimal Values.**

| Model | Hyperparameter | Search Range / Values Tested | Optimal Value Selected |
| --- | --- | --- | --- |
| Gradient Boosted Trees | number_of_trees | [20, 30, 50, 60, 70, 80, 100] | 20 |
|  | maximal_depth | [5, 10, 20, 30, 50] | 5 |
|  | learning_rate | [0.01, 0.05, 0.1, 0.2] | 0.1 |
| Decision Tree | criterion | [gain_ratio, information_gain, gini_index, accuracy, least_square] | information_gain |
|  | maximal_depth | [3, 5, 7, 10, 15, 20, 30, 40, 50] | 3 |
|  | minimal_size_for_split | [2, 4, 6, 8, 10] | 2 |
| Random Forest | number_of_trees | [20, 40, 60, 80, 100, 120] | 20 |
|  | maximal_depth | [0;100.0;10;linear] | 50 |
|  | voting_strategy | [confidence vote, majority vote] | confidence vote |
|  | criterion | [gain_ratio, information_gain, gini_index, accuracy, least_square] | gini_index |
| Decision Stump | criterion | [gain_ratio, information_gain, gini_index, accuracy] | gain_ratio |
|  | minimal_leaf_size | [1.0;100.0;10;linear] | 1 |
| Random Tree | criterion | [gain_ratio, information_gain, gini_index, accuracy] | gain_ratio |
|  | minimal_size_for_split | [2, 4, 8, 10, 15] | 10 |
|  | number_of_prepruning_alternatives | [3, 5, 7, 10] | 7 |
|  | maximal_depth | [5, 7, 10, 20, 30, 40, 50] | 40 |
| Note: Values such as [0;100.0;10;linear] represent the standard RapidMiner format for a range, indicating values between 0 and 100 tested in 10 linear steps. | | | |
