# Supplementary material for: From data to decisions: Predicting inpatient burn mortality with advanced classification models
Source: PLoS One. 2026 Jan 2;21(1):e0338564. doi: 10.1371/journal.pone.0338564 (PMC12758681; doi:10.1371/journal.pone.0338564)
Supplement: S3 Table — Ranking of features using multiple weighting criteria. (DOCX) [file pone.0338564.s003.docx]

## **S3 Table. Exploratory analysis of feature importance using multiple weighting criteria.**

| **Item** | **GINI** | **Correlation** | **Information Gain** | **Information Gain Ratio** | **Chi Squared Statistic** | **Uncertainty** | **Tree Importance** |
| --- | --- | --- | --- | --- | --- | --- | --- |
| 1 | Qualitative Classification of ABSI | Score of ABSI | Qualitative Classification of ABSI | Score of ABSI | Score of ABSI | Qualitative Classification of ABSI | Score of ABSI |
| 2 | Score of ABSI | Burn-Related Information: TBSA | Burn-Related Information: Third-Degree Burns | Burn-Related Information: TBSA | Qualitative Classification of ABSI | Burn-Related Information: TBSA | Burn-Related Information: Third-Degree Burns |
| 3 | Burn-Related Information: Third-Degree Burns | Qualitative Classification of ABSI | Burn-Related Information: TBSA | Qualitative Classification of ABSI | Burn-Related Information: Third-Degree Burns | Score of ABSI | Qualitative Classification of ABSI |
| 4 | Burn-Related Information: Total Burn Surface Area | Symptom of Inhalation Injury: Low Oxygen Saturation | Score of ABSI | Burn-Related Information: Third-Degree Burns | Burn-Related Information: TBSA | Burn-Related Information: Third-Degree Burns | Burn-Related Information: TBSA |
| 5 | cause of Inhalation Injury: Third-Degree Burn of the Neck and Anterior Torso | cause of Inhalation Injury: Heat Inhalation | cause of Inhalation Injury: Third-Degree Burn of the Neck and Anterior Torso | cause of Inhalation Injury: Third-Degree Burn of the Neck and Anterior Torso | cause of Inhalation Injury: Third-Degree Burn of the Neck and Anterior Torso | cause of Inhalation Injury: Third-Degree Burn of the Neck and Anterior Torso | Symptom of Inhalation Injury: Low Oxygen Saturation |
| 6 | cause of Inhalation Injury: Heat Inhalation | Paraclinical Indicators: HCT | cause of Inhalation Injury: Heat Inhalation | cause of Inhalation Injury: Heat Inhalation | cause of Inhalation Injury: Heat Inhalation | cause of Inhalation Injury: Heat Inhalation | Paraclinical Indicators: HCT |
| 7 | Symptom of Inhalation Injury: Low Oxygen Saturation | Burn Area: Posterior Torso | Symptom of Inhalation Injury: Low Oxygen Saturation | cause of Inhalation Injury: Face Burn | Symptom of Inhalation Injury: Low Oxygen Saturation | cause of Inhalation Injury: Face Burn | Burn-Related Information: Second-Degree Burns |
| 8 | cause of Inhalation Injury: Face Burn | Paraclinical Indicators: Hemoglobin | cause of Inhalation Injury: Face Burn | Burn Area: Posterior Torso | cause of Inhalation Injury: Face Burn | Symptom of Inhalation Injury: Low Oxygen Saturation | Paraclinical Indicators: BUN |
| 9 | Symptom of Inhalation Injury: Hoarseness | Burn Area: Anterior Torso | Symptom of Inhalation Injury: Hoarseness | Symptom of Inhalation Injury: Low Oxygen Saturation | Symptom of Inhalation Injury: Hoarseness | Burn Area: Posterior Torso | Paraclinical Indicators: Albumin |
| 10 | Admission and Referral Data: Braden Score | Admission and Referral Data: Morse (Humpty Dumpty) Score | Symptom of Inhalation Injury: Cough | Pre-hospital Care: Intubation | Admission and Referral Data: Braden Score | Admission and Referral Data: Braden Score | Paraclinical Indicators: Hemoglobin |
| 11 | Symptom of Inhalation Injury: Cough | Symptom of Inhalation Injury: Hoarseness | Symptom of Inhalation Injury: Shortness of Breath | Admission and Referral Data: Braden Score | Symptom of Inhalation Injury: Cough | Paraclinical Indicators: Albumin | Admission and Referral Data: Transport Mode |
| 12 | Symptom of Inhalation Injury: Shortness of Breath | Pre-hospital Care: Intubation | Symptom of Inhalation Injury: Burnt Hair | Burn Area: Anterior Torso | Symptom of Inhalation Injury: Shortness of Breath | Symptom of Inhalation Injury: Hoarseness | cause of Inhalation Injury: Heat Inhalation |
| 13 | Symptom of Inhalation Injury: Burnt Hair | Symptom of Inhalation Injury: Shortness of Breath | Admission and Referral Data: Braden Score | Paraclinical Indicators: BUN | Symptom of Inhalation Injury: Burnt Hair | Paraclinical Indicators: HCT | cause of Inhalation Injury: Face Burn |
| 14 | Paraclinical Indicators: HCT | Admission and Referral Data: Braden Score | Burn Area: Posterior Torso | Paraclinical Indicators: Albumin | Paraclinical Indicators: HCT | Symptom of Inhalation Injury: Burnt Hair | cause of Inhalation Injury: Third-Degree Burn of the Neck and Anterior Torso |
| 15 | Paraclinical Indicators: Albumin | Pre-hospital Care: Nasogastric Tube | Paraclinical Indicators: HCT | Paraclinical Indicators: HCT | Paraclinical Indicators: Albumin | Symptom of Inhalation Injury: Shortness of Breath | Admission and Referral Data: Morse (Humpty Dumpty) Score |
| 16 | Paraclinical Indicators: Hemoglobin | Symptom of Inhalation Injury: Cough | Paraclinical Indicators: Albumin | Symptom of Inhalation Injury: Burnt Hair | Burn Area: Posterior Torso | Symptom of Inhalation Injury: Cough | Demographic: Age |
| 17 | Paraclinical Indicators: BUN | Admission and Referral Data: Transport Mode | Paraclinical Indicators: Hemoglobin | Symptom of Inhalation Injury: Shortness of Breath | Paraclinical Indicators: Hemoglobin | Burn Area: Anterior Torso | Demographic: Ethnicity |
| 18 | Burn-Related Information: Second-Degree Burns | Inhalation Injury? | Burn-Related Information: Second-Degree Burns | Symptom of Inhalation Injury: Hoarseness | Paraclinical Indicators: BUN | Paraclinical Indicators: BUN | Burn-Related Information: Burn Degree |
| 19 | Burn-Related Information: Burn Degree | Burn Care in ER: Nasogastric Tube Placement | Admission and Referral Data: Transport Mode | Pre-hospital Care: Nasogastric Tube | Burn-Related Information: Second-Degree Burns | Paraclinical Indicators: Hemoglobin | Demographic: Marital Status |
| 20 | Paraclinical Indicators: Creatinine | Pre-hospital Care: Urinary Catheter | Burn Area: Anterior Torso | Symptom of Inhalation Injury: Cough | Burn-Related Information: Burn Degree | Pre-hospital Care: Intubation | Admission and Referral Data: Braden Score |
| 21 | Admission and Referral Data: Transport Mode | Admission and Referral Data: Wells Score | Burn-Related Information: Burn Degree | Burn Area: Hands | Burn Area: Anterior Torso | Burn-Related Information: Second-Degree Burns | Paraclinical Indicators: Creatinine |
| 22 | Admission and Referral Data: Morse (Humpty Dumpty) Score | Burn Area: Feet | Paraclinical Indicators: BUN | Paraclinical Indicators: Hemoglobin | Paraclinical Indicators: Creatinine | Burn-Related Information: Burn Degree | Burn Area: Posterior Torso |
| 23 | Burn-Related Information: Burn Type | Burn-Related Information: Burn Type | Admission and Referral Data: Morse (Humpty Dumpty) Score | Burn Care in ER: Nasogastric Tube Placement | Admission and Referral Data: Transport Mode | Pre-hospital Care: Nasogastric Tube | Demographic: Weight |
| 24 | Burn Type: Type of Hot Liquid | Paraclinical Indicators: Albumin | Burn-Related Information: Burn Type | Burn Area: Feet | Admission and Referral Data: Morse (Humpty Dumpty) Score | Admission and Referral Data: Morse (Humpty Dumpty) Score | Symptom of Inhalation Injury: Hoarseness |
| 25 | Medical History: COVID-19 Infection | Burn Type: Type of Hot Liquid | Paraclinical Indicators: Creatinine | Burn-Related Information: Fourth-Degree Burns | Pre-hospital Care: Intubation | Admission and Referral Data: Transport Mode | Demographic: Education Level |
| 26 | Admission and Referral Data: Wells Score | Paraclinical Indicators: FBS | Burn Type: Type of Hot Liquid | Inhalation Injury? | Burn-Related Information: Burn Type | Burn Area: Feet | Demographic: BMI (years > 15 ) |
| 27 | Paraclinical Indicators: FBS | Post-ER: Patient Status | Admission and Referral Data: Wells Score | Burn-Related Information: Burn Degree | Pre-hospital Care: Nasogastric Tube | Inhalation Injury? | Paraclinical Indicators: FBS |
| 28 | Paraclinical Indicators: Potassium | Burn-Related Information: Burn Degree | Pre-hospital Care: Intubation | Admission and Referral Data: Morse (Humpty Dumpty) Score | Burn Type: Type of Hot Liquid | Burn-Related Information: Burn Type | Medical History: COVID-19 Infection |
| 29 | Demographic: Age | Demographic: Gender | Inhalation Injury? | Admission and Referral Data: Wells Score | Medical History: COVID-19 Infection | Admission and Referral Data: Wells Score | Demographic: Gender |
| 30 | Demographic: Education Level | Demographic: Under 6 Years Old? | Burn Area: Feet | Burn-Related Information: Second-Degree Burns | Admission and Referral Data: Wells Score | Paraclinical Indicators: Creatinine | Symptom of Inhalation Injury: Shortness of Breath |
| ***ABSI:*** *Abbreviated Burn Severity Index;* ***TBSA:*** *Total Burn Surface Area* | | | | | | | |
