# Supplementary material for: From data to decisions: Predicting inpatient burn mortality with advanced classification models
Source: PLoS One. 2026 Jan 2;21(1):e0338564. doi: 10.1371/journal.pone.0338564 (PMC12758681; doi:10.1371/journal.pone.0338564)
Supplement: S4 Table — Aggregated confusion matrices for GBT, RF, DT, DS, and RT models. (DOCX) [file pone.0338564.s004.docx]

## **S4 Table. Confusion matrix of the Final Models.**

| **GBT** | **True Survived** | **True Deceased** | **RF** | **True Survived** | **True Deceased** | **RT** | **True Survived** | **True Deceased** |
| --- | --- | --- | --- | --- | --- | --- | --- | --- |
| Pred. Survived | 505 | 33 | Pred. Survived | 505 | 34 | Pred. Survived | 490 | 61 |
| Pred. Deceased | 13 | 111 | Pred. Deceased | 13 | 110 | Pred. Deceased | 28 | 83 |
| **DT** | True Survived | True Deceased | **DS** | True Survived | True Deceased |  | | |
| Pred. Survived | 502 | 41 | Pred. Survived | 515 | 77 |  |  |  |
| Pred. Deceased | 16 | 103 | Pred. Deceased | 3 | 67 |  |  |  |
