# Supplementary material for: From data to decisions: Predicting inpatient burn mortality with advanced classification models
Source: PLoS One. 2026 Jan 2;21(1):e0338564. doi: 10.1371/journal.pone.0338564 (PMC12758681; doi:10.1371/journal.pone.0338564)
Supplement: S5 Table — Statistical comparison of accuracy between models. (DOCX) [file pone.0338564.s005.docx]

## **S5 Table. Full Pairwise T-test p-values for Model Accuracy.**

| **Model** |  | **GBT** | **DT** | **RF** | **DS** | **RT** | **GBT** | **RF** | **GBT** | **RF** |
| --- | --- | --- | --- | --- | --- | --- | --- | --- | --- | --- |
|  | **Methodological Condition** | Continuous + GLM | | | | | Continuous + Mean/Fixed Value Imputation | | Categorical Variables + GLM | |
| **GBT** | Continuous + GLM | - | 0.104 | 0.896 | 0.003 | 0.001 | 0.762 | 0.579 | 0.451 | 0.55 |
| **DT** |  | 0.104 | - | 0.197 | 0.032 | 0.009 | 0.197 | 0.344 | 0.38 | 0.31 |
| **RF** |  | 0.896 | 0.197 | - | 0.006 | 0.002 | 0.892 | 0.712 | 0.6 | 0.696 |
| **DS** |  | 0.003 | 0.032 | 0.006 | - | 0.499 | 0.005 | 0.009 | 0.009 | 0.008 |
| **RT** |  | 0.001 | 0.009 | 0.002 | 0.499 | - | 0.002 | 0.003 | 0.003 | 0.002 |
| **GBT** | Continuous + Mean/Fixed Value Imputation | 0.762 | 0.197 | 0.892 | 0.005 | 0.002 | - | 0.791 | 0.665 | 0.776 |
| **RF** |  | 0.579 | 0.344 | 0.712 | 0.009 | 0.003 | 0.791 | - | 0.89 | 1 |
| **GBT** | Categorical Variables + GLM | 0.451 | 0.38 | 0.6 | 0.009 | 0.003 | 0.665 | 0.89 | - | 0.883 |
| **RF** |  | 0.55 | 0.31 | 0.696 | 0.008 | 0.002 | 0.776 | 1 | 0.883 | - |
| **Note:** Values with colored background are smaller than alpha=0.05 which indicates a probably significant difference between the actual mean values. | | | | | | | | | | |
