# Supplementary material for: From data to decisions: Predicting inpatient burn mortality with advanced classification models
Source: PLoS One. 2026 Jan 2;21(1):e0338564. doi: 10.1371/journal.pone.0338564 (PMC12758681; doi:10.1371/journal.pone.0338564)
Supplement: S6 Table — Statistical comparison of AUC between models. (DOCX) [file pone.0338564.s006.docx]

## **S6 Table. Full Pairwise T-test p-values for the AUC Metric.**

| **Model** |  | **GBT** | **DT** | **RF** | **DS** | **RT** | **GBT** | **RF** | **GBT** | **RF** |
| --- | --- | --- | --- | --- | --- | --- | --- | --- | --- | --- |
|  | **Methodological Condition** | Continuous + GLM | | | | | Continuous + Mean/Fixed Value Imputation | | Categorical Variables + GLM | |
| **GBT** | Continuous + GLM |  | 0 | 0.393 | 0 | 0 | 0.404 | 0.859 | 0.638 | 0.309 |
| **DT** |  | 0 | - | 0.001 | 0 | 0.002 | 0.001 | 0 | 0.001 | 0.001 |
| **RF** |  | 0.393 | 0.001 | - | 0 | 0 | 0.949 | 0.397 | 0.932 | 0.751 |
| **DS** |  | 0 | 0 | 0 | - | 0.398 | 0 | 0 | 0 | 0 |
| **RT** |  | 0 | 0.002 | 0 | 0.398 | - | 0 | 0 | 0 | 0 |
| **GBT** | Continuous + Mean/Fixed Value Imputation | 0.404 | 0.001 | 0.949 | 0 | 0 | - | 0.399 | 0.898 | 0.808 |
| **RF** |  | 0.859 | 0 | 0.397 | 0 | 0 | 0.399 | - | 0.588 | 0.312 |
| **GBT** | Categorical Variables + GLM | 0.638 | 0.001 | 0.932 | 0 | 0 | 0.898 | 0.588 | - | 0.752 |
| **RF** |  | 0.309 | 0.001 | 0.751 | 0 | 0 | 0.808 | 0.312 | 0.752 | - |
| **Note:** Values with colored background are smaller than alpha=0.05 which indicates a probably significant difference between the actual mean values. | | | | | | | | | | |
