# Supplementary material for: From data to decisions: Predicting inpatient burn mortality with advanced classification models
Source: PLoS One. 2026 Jan 2;21(1):e0338564. doi: 10.1371/journal.pone.0338564 (PMC12758681; doi:10.1371/journal.pone.0338564)
Supplement: S7 Table — Statistical comparison of Brier scores between models. (DOCX) [file pone.0338564.s007.docx]

## **S7 Table. Full Pairwise T-test p-values for the Brier Score.**

| **Model** |  | **GBT** | **DT** | **RF** | **DS** | **RT** | **GBT** | **RF** | **GBT** | **RF** |
| --- | --- | --- | --- | --- | --- | --- | --- | --- | --- | --- |
|  | **Methodological Condition** | Continuous + GLM | | | | | Continuous + Mean/Fixed Value Imputation | | Categorical Variables + GLM | |
| **GBT** | Continuous + GLM | - | 0.107 | 0.620 | 0.001 | 0.000 | 0.618 | 0.443 | 0.443 | 0.383 |
| **DT** |  | 0.107 | - | 0.146 | 0.011 | 0.004 | 0.224 | 0.264 | 0.453 | 0.415 |
| **RF** |  | 0.620 | 0.146 | - | 0.001 | 0.000 | 0.929 | 0.698 | 0.643 | 0.577 |
| **DS** |  | 0.001 | 0.011 | 0.001 | - | 0.771 | 0.001 | 0.001 | 0.004 | 0.003 |
| **RT** |  | 0.000 | 0.004 | 0.000 | 0.771 | - | 0.000 | 0.000 | 0.001 | 0.001 |
| **GBT** | Continuous + Mean/Fixed Value Imputation | 0.618 | 0.224 | 0.929 | 0.001 | 0.000 | - | 0.816 | 0.727 | 0.683 |
| **RF** |  | 0.443 | 0.264 | 0.698 | 0.001 | 0.000 | 0.816 | - | 0.856 | 0.821 |
| **GBT** | Categorical Variables + GLM | 0.443 | 0.453 | 0.643 | 0.004 | 0.001 | 0.727 | 0.856 | - | 0.988 |
| **RF** |  | 0.383 | 0.415 | 0.577 | 0.003 | 0.001 | 0.683 | 0.821 | 0.988 | - |
| **Note:** Values with colored background are smaller than alpha=0.05 which indicates a probably significant difference between the actual mean values. | | | | | | | | | | |
