# Supplementary material for: From data to decisions: Predicting inpatient burn mortality with advanced classification models
Source: PLoS One. 2026 Jan 2;21(1):e0338564. doi: 10.1371/journal.pone.0338564 (PMC12758681; doi:10.1371/journal.pone.0338564)
Supplement: S8 Table — (DOCX) [file pone.0338564.s008.docx]

## **S8 Table. Statistical comparison (p-values) of model performance with and without the ABSI feature.**

| Model | Comparison | Accuracy (p-value) | AUC (p-value) | Brier Score (p-value) |
| --- | --- | --- | --- | --- |
| GBT | With ABSI vs. Without ABSI | 0.504 | 0.53 | 0.523 |
| RF | With ABSI vs. Without ABSI | 0.775 | 0.822 | 0.827 |
