# Supplementary material for: From data to decisions: Predicting inpatient burn mortality with advanced classification models
Source: PLoS One. 2026 Jan 2;21(1):e0338564. doi: 10.1371/journal.pone.0338564 (PMC12758681; doi:10.1371/journal.pone.0338564)
Supplement: S1 Checklist — Completed checklist demonstrating adherence to reporting guidelines for prediction model development and validation. (DOCX) [file pone.0338564.s009.docx]

## **S1 Checklist. TRIPOD Checklist: Prediction Model Development and Validation.**

| Item # | Topic | Location to write in Checklist |
| --- | --- | --- |
| Title |  |  |
| 1 | Title | Page 1 - Title Page |
| Abstract |  |  |
| 2 | Abstract | Page 2 - Abstract |
| Introduction |  |  |
| 3a | Background | Page 3 - Introduction (Paragraphs 1-3) |
| 3b | Objectives | Page 4 - Introduction (Last Paragraph) |
| Methods |  |  |
| 4a | Source of data | Page 5 - Methods: Dataset Description |
| 4b | Dates | Page 5 - Methods: Dataset Description (12/04/2022 to 08/10/2023) |
| 5a | Participants (selection) | Page 5 - Methods: Dataset Description |
| 5b | Participants (treatment) | Page 5 - Methods: Dataset Description (Inpatient burn registry) |
| 6a | Outcome definition | Page 5 - Methods: Dataset Description (Final outcome: Died/Alive) |
| 6b | Outcome blindness | N/A (Retrospective registry data) |
| 7a | Predictors definition | Page 5 - Methods: Dataset Description & S1 Table (Supporting Information) |
| 7b | Predictors blindness | N/A (Retrospective study) |
| 8 | Sample size | Page 5 - Methods: Dataset Description (n=651) |
| 9 | Missing data | Page 6 - Methods: Handling Missing Values |
| 10a | Predictors handling | Page 6 - Methods: Initial Feature Engineering and Selection |
| 10b | Model type | Page 7 - Methods: Model Development and Hyperparameter Tuning |
| 10c | Model building procedures | Page 7 & 8 - Methods: Model Development (Nested CV & Grid Search) |
| 10d | Internal validation | Page 9 - Methods: Model Performance Evaluation (10-fold Stratified CV) |
| 10e | Temporal/External validation | Page 10 - Methods: Temporal Validation and Sensitivity Analyses |
| 11 | Risk groups | N/A (Individual probabilities used) |
| 12 | Development vs. Validation | Page 10 - Methods: Temporal Validation (Split description) |
| Results |  |  |
| 13a | Participant flow | Page 12 - Results: Study Population & Fig 1 |
| 13b | Participant characteristics | Page 12 - Results: Study Population & S1 Table (Supporting Information) |
| 13c | Missing data summary | Page 6 - Methods: Handling Missing Values & S1 Table (Supporting Information) |
| 14a | Model specification | Page 8 & 13 - Results: Main Model Performance & Table 1 (Hyperparameters) |
| 14b | Model performance (Dev) | Page 13 - Results: Main Model Performance (Table 3, Fig 2) |
| 15a | Model performance (Val) | Page 15 - Results: Temporal Validation (Table 5, Fig 5) |
| 15b | Model update | N/A (Model was frozen, not updated) |
| 16 | Model comparison | Page 14 - Results: Statistical Comparison of Models (S5-S8 Tables (Supporting Information)) |
| Discussion |  |  |
| 17 | Limitations | Page 21 - Discussion: Strengths and Limitations |
| 18 | Interpretation | Page 19 - Discussion (Paragraphs 1-5) |
| 19a | Implications | Page 20 - Discussion: Clinical Implications |
| 19b | Future research | Page 22 - Conclusion (Last paragraph) |
| Other |  |  |
| 20 | Supplementary info | Supporting Information (S1-S8 Tables, Fig 4-5) |
| 21 | Funding | Funding (None) |
| 22 | Data & Code | Availability of Data and Materials |
