# Supplementary material for: From data to decisions: Predicting inpatient burn mortality with advanced classification models
Source: PLoS One. 2026 Jan 2;21(1):e0338564. doi: 10.1371/journal.pone.0338564 (PMC12758681; doi:10.1371/journal.pone.0338564)
Supplement: Attachment — Submitted filename: Response to Reviewers-14040321.docx [file pone.0338564.s011.docx]

**PONE-D-25-02698** **From Data to Decisions: Predicting Inpatient Burn Mortality with Advanced Classification Models** **PLOS ONE**

**June 11, 2025**

Dear Dr. Erfan Taherifard, Academic Editor, and esteemed Reviewers,

Thank you for providing us with the opportunity to revise our manuscript. We are grateful for the thoughtful and highly constructive feedback from both the editor and the reviewers. We believe the manuscript has been substantially strengthened by incorporating these suggestions. We have performed a comprehensive new analysis, including more robust validation methods, sensitivity analyses, and a deeper interpretation of the results, which has led to a more nuanced and impactful manuscript.

Below, we provide a point-by-point response to the comments. All changes in the manuscript have been highlighted using the "Track Changes" feature.

**Editor’s Comments**

**Journal Requirement 1: Style Requirements & File Naming**

- **Response:** We have carefully reviewed the PLOS ONE style templates and have revised the manuscript and file naming to ensure full compliance with the journal's formatting requirements, including the use of Fig 1 and bracketed citations [1].

**Journal Requirement 2 & 4: Code and Data Availability**

- **Response:** We thank the editor for this important point. We have updated our Data Availability Statement to be fully compliant with PLOS ONE’s policy. While the clinical dataset cannot be publicly shared due to ethical and ownership restrictions, the complete RapidMiner processes used for the analysis have been deposited to the Zenodo repository and are accessible via DOI: 10.5281/zenodo.15639850.

**Journal Requirement 3: Ethics Statement & Data Access**

- **Response:** We confirm that the ethics statement appears only in the Methods section. We have also updated the Availability of Data and Materials section to provide a detailed explanation of the data access restrictions and have included the formal contact information for the Isfahan University of Medical Sciences Ethics Committee for any access requests by qualified researchers.

**Reviewer #1 Comments**

**Comment 1.1: Abstract Lacks Detail**

- **Response:** We agree. The abstract has been completely revised to include the final sample size (n=651), a description of the new methodology, the key performance metrics of the best model (including Brier Score), and the main finding regarding concept drift.

**Comment 1.2: Citing Original Sources**

- **Response:** We have reviewed all references and replaced secondary citations with primary sources, specifically for the WHO statistic.

**Comment 1.3: Contextual Relevance (Iran) and Complications**

- **Response:** The Introduction now focuses on the burden of burn injuries in Iran and includes the suggested reference by Soltany, A., et al. (2024).

**Comment 1.4 & 1.7: Underdeveloped Discussion and Conclusion Tone**

- **Response:** The Discussion and Conclusion sections have been entirely rewritten to provide a deep interpretation of the new, more robust findings, including clinical implications and concept drift, with a more nuanced and cautious tone.

**Comment 1.5: Clearer Articulation of Limitations**

- **Response:** Thank you. The Limitations section has been expanded to be more comprehensive. Furthermore, to clarify the initial data exclusion, we note that the study began with 662 valid patient records after an initial 415 empty or test entries were removed from the registry. These were not actual patient records with missing data, but rather system test entries, and thus do not introduce selection bias. The exclusion of the 11 patient records with missing outcomes from the final cohort is now clearly detailed in the STROBE diagram (Figure 1).

**Comment 1.6: Future Directions**

- **Response:** Future research directions in the Conclusion have been expanded upon to be more comprehensive.

**Reviewer #2 Comments**

**Comment 2.1: Novelty and Differentiation**

- **Response:** The Introduction now explicitly differentiates our work from traditional prognostic scores (ABSI/Baux) and prior machine learning studies by highlighting our use of a more comprehensive dataset and a more rigorous validation framework.

**Comment 2.2: Data Preprocessing Transparency**

- **Response:** Thank you for this valuable comment. We have enhanced transparency in several ways:
  - **Analysis of Excluded Data:** We would like to clarify that the initial dataset contained 1,077 entries; however, 415 of these records were entirely empty or had only minimal metadata, with all clinical fields left blank. Based on our coordination with the hospital registry team, these entries were likely generated during the early testing phase of the registry system and do not reflect actual patient admissions. As such, they do not represent systematically missing data but rather non-clinical test records. Consequently, we considered 662 valid patient records as the actual starting point of our analysis. This is now clarified in the Methods section. The STROBE diagram (Figure 1) further details the exclusion of the 11 patients with missing outcomes from this cohort.
  - **Imputation and Categorization:** Our primary analysis now uses a robust GLM Imputation method on continuous variables. We have also performed sensitivity analyses (Table 6) to assess the impact of both the imputation method and the use of categorized variables, as requested.

**Comment 2.3: Methodological Rigor**

- **Response:** The methodological rigor has been substantially enhanced:
  - The entire analysis now uses **10-fold stratified cross-validation**.
  - We performed **hyperparameter tuning using a grid search**, with the process described in the Methods and full details provided in **S2 Table**.
  - We have conducted **paired t-tests** to statistically compare model performances. A summary of these findings is now in the Results section, with full details in **S4-S6 Tables**.

**Comment 2.4: External Validation and Generalizability**

- **Response:** We thank the reviewer for this crucial point. While a true external validation using data from another center is the gold standard for assessing generalizability, we were unable to perform this for the current study due to significant logistical and regulatory challenges. Accessing clinical data in Iran is subject to strict patient privacy and confidentiality laws, and the process of obtaining ethical approvals and data use agreements from multiple institutions is lengthy and complex. Furthermore, there is a high probability of dataset mismatch, where the features collected in an external registry may not align with our own, making a direct validation unfeasible.
- However, to address the core of the reviewer's concern about model stability over time, we performed a rigorous **temporal validation**. The results of this analysis, which revealed significant concept drift, are presented in **Table 5** and discussed extensively as a key finding. We believe this temporal validation serves as a strong proxy for external validation.

**Comment 2.5: Clinical Interpretability and Calibration**

- **Response:** We have added a dedicated **"Clinical Implications"** section to the Discussion. Furthermore, we have now reported the **Brier Score** for all models (**Table 4**) to explicitly assess and discuss model calibration.

**Comment 2.6: Reporting Guidelines (TRIPOD/STROBE)**

- **Response:** We have added a **STROBE diagram (Figure 1)**, and the manuscript has been structured to better align with **TRIPOD** principles by detailing model development, validation, calibration, and performance evaluation. The **Ethics Approval** section has been updated with full details on anonymization and consent waivers.

**Comment 2.7 & 2.8: Clarity and Terminology**

- **Response:** The manuscript has been thoroughly revised for clarity. **Figure 2 (ROC curve)** has been improved with explicit labels. All tables have been redesigned, and terminology has been standardized throughout the manuscript.

We thank the editor and reviewers again for their time and valuable insights. We hope the revised manuscript is now suitable for publication in PLOS ONE.

Sincerely,

Yasin Sabet Kouhanjani (on behalf of all authors)
