# Supplementary material for: From data to decisions: Predicting inpatient burn mortality with advanced classification models
Source: PLoS One. 2026 Jan 2;21(1):e0338564. doi: 10.1371/journal.pone.0338564 (PMC12758681; doi:10.1371/journal.pone.0338564)
Supplement: Attachment — Submitted filename: Response to Reviewers-14040608.docx [file pone.0338564.s012.docx]

**PONE-D-25-02698R1**

**From Data to Decisions: Predicting Inpatient Burn Mortality with Advanced Classification Models**

**PLOS ONE**

August 30, 2025

Dear Dr. Amirreza Khalaji, Academic Editor, and esteemed Reviewers,

Thank you for the thorough review of our manuscript and for providing us with the opportunity to submit a revised version. We are grateful for the insightful and highly constructive feedback, which we believe has substantially strengthened our work. We have carefully addressed every point raised, leading to a more robust, transparent, and impactful manuscript.

First and foremost, we would like to notify the editor and reviewers of an important correction made during our verification process for this revision. Upon re-running our temporal validation analysis to ensure full reproducibility, our new and consistent results revealed that the models demonstrated much stronger temporal stability than was presented in the initial submission. We believe these new, reproducible results are more accurate and reflect the models' true, robust performance. Consequently, we have **updated Table 5** and adjusted the narrative in the **Abstract, Discussion, and Conclusion** to align with this significant new finding.

Below, we provide a point-by-point response to each of the comments. All changes in the manuscript have been highlighted using the "Track Changes" feature for your convenience.

**Reviewer #1**

**Comment:** *“The authors have thoroughly addressed all comments and concerns raised in the previous round of review... I believe the manuscript has significantly improved and now meets the standards for publication in its current form.”*

- **Response:** We sincerely thank Reviewer #1 for their positive feedback and for recognizing the effort put into the previous revision. We are grateful for their endorsement.

**Reviewer #2**

**Comment 1: Reframing the Manuscript's Core Contribution.** *The central theme must shift... The primary story is no longer "we built a good model," but rather, "we demonstrate the critical instability of a seemingly good model when tested over time."*

- **Response:** We thank the reviewer for this crucial and insightful feedback. We agree that our new finding regarding the model’s temporal performance has fundamentally reframed the manuscript's core message. As noted in our introductory remarks, upon re-running our analyses, we found that the model is, in fact, remarkably stable. We have therefore rewritten the manuscript's narrative to reflect this new, more accurate finding. The new story is now one of a robust model that demonstrates strong temporal stability, albeit with a clinically important trade-off (a decrease in sensitivity). This new, more positive, and nuanced narrative has been consistently integrated throughout the revised **Abstract**, **Introduction**, **Discussion**, and **Conclusion**.

**Comment 2: A Deeper and More Critical Analysis of Clinical Risks.** *Highlight the Danger of Decreased Specificity... Correct the Misunderstanding of Validation Methods.*

- **Response:** This comment was based on our previous, incorrect temporal validation results which showed a large drop in specificity. Our new, reproducible results show the opposite: specificity remains excellent, while sensitivity decreases. We have completely rewritten the **"Clinical Implications"** section of the Discussion to analyze the real-world risks associated with this **decreased sensitivity**, namely the danger of false reassurances and missed opportunities for intervention in high-risk patients. We also thank the reviewer for pointing out the methodological misunderstanding. We have revised the **"Strengths and Limitations"** section to state clearly that temporal validation assesses stability at one site and is **not a substitute for external validation**.

**Comment 3: Ensuring Logical Consistency in the Conclusion.** *The conclusion currently lacks logical consistency... The final take-home message should not be about the model's "power".*

- **Response:** We agree completely. The **Conclusion** has been entirely rewritten. The new conclusion now leads with the key finding of strong temporal stability but immediately follows with the critical caveat of decreased sensitivity, emphasizing that even stable models require a robust governance framework of continuous monitoring and recalibration for safe clinical deployment.

**Reviewer #3**

**Comment 1: Dataset Description.** *(Requests for more detail on observations, features, longitudinal data, temporal validation, etc.)*

- **Response:** We have thoroughly revised the **"Dataset Description"** and **"Data Preprocessing"** sections in Methods to address all these points. We now explicitly state the number of patients (651) and final features (93), confirm that each patient is a single observation and that no longitudinal data were used, clarify the one-hot encoding of composite features, and provide a clear definition of the temporal validation cohorts in the **"Temporal Validation and Sensitivity Analyses"** subsection.

**Comment 2: Removed Features in the Preprocessing Steps.**

- **Response:** We have expanded the **"Initial Feature Selection and Reduction"** subsection to provide a clearer rationale for feature exclusion, including an example of a feature removed due to class imbalance (a specific comorbidity).

**Comment 3: Missing Values.** *(Requests for more detail on missingness, GLM justification, 85% threshold, and sequence of steps.)*

- **Response:** We have completely rewritten the relevant sections to address these points. The **"Handling Missing Values"** subsection now: (1) Provides an overall percentage of features with missing values. (2) Clarifies that the outcome variable (label) was excluded from the imputation process. (3) Justifies the use of GLM by noting it was in response to a previous review and our sensitivity analysis confirmed it offered a slight performance improvement. (4) Justifies the 85% threshold as a pragmatic balance. (5) Clarifies the sequence of operations (high-missingness removal followed by imputation).

**Comment 4: Machine Learning Models.** *(Requests for rationale for tree-based models and a table for hyperparameters.)*

- **Response:** We have added a new introductory paragraph to the **"Model Development and Hyperparameter Tuning"** subsection that articulates our rationale for focusing on tree-based models. We have also followed the reviewer's excellent suggestion and moved the optimal hyperparameters from the text into a new, clear summary table (**Table 1**).

**Comment 5: Validation.** *(Requests for clearer temporal validation process, definition of stability, and explanation of Brier Score.)*

- **Response:** The **"Temporal Validation"** subsection has been rewritten for clarity, explicitly stating the cohorts are non-overlapping. "Model stability" is now defined at the start of this section. We have also expanded the explanation of the **Brier Score** in the **"Model Performance Evaluation"** subsection to describe its clinical relevance for assessing calibration.

**Comment 6: Feature Importance.** *(Suggestion to add SHAP and discuss alignment with clinical knowledge.)*

- **Response:** We thank the reviewer for this valuable suggestion. As explained in the **"Strengths and Limitations"** section, we were unable to implement SHAP analysis due to technical limitations of our analytical software. However, to address the core of this comment, we have replaced our previous complex table with a clear visualization (**Figure 3**) of the feature importance from our best model (GBT). Furthermore, we have added a new paragraph to the **Discussion** that explicitly discusses how these top-ranked features (ABSI, TBSA, etc.) align with established clinical knowledge.

**Reviewer #4**

**Comment 1 & 2: Data Preprocessing, Imputation, and Risk of Overfitting.**

- **Response:** We have significantly enhanced the transparency of our methods. The **"Handling Missing Values"** section now explicitly states that the imputation pipeline was nested within the cross-validation folds to prevent data leakage. The **"Model Development and Hyperparameter Tuning"** section now clarifies that we used a nested validation procedure. We have also added a discussion of the risk of **overfitting** in the **"Strengths and Limitations"** section.

**Comment 3: Evaluation Metrics and Class Imbalance.**

- **Response:** We agree that this is a critical point. We have added a new paragraph to the **"Main Model Performance"** subsection in the Results, which explicitly discusses the key class-wise metrics (sensitivity, specificity, and PPV) for our best-performing model in the main text.

**Comment 4: Use of Composite Scores (e.g., ABSI) as Features.**

- **Response:** We have performed the requested sensitivity analysis. The process is now described in the **"Temporal Validation and Sensitivity Analyses"** subsection of the Methods, and the results (which show the model's performance remains strong without ABSI) are presented in **Table 7** and discussed in both the **Results** and **Discussion** sections.

**Comment 5: Interpretation of Feature Importance.**

- **Response:** We have added a paragraph to the **Discussion** that confirms the main predictors align with known clinical risk factors and explicitly states that no entirely novel predictors were identified, framing the result as a reinforcement of clinical knowledge.

**Comment 6 & 7: External Validation and Handling of Concept Drift.**

- **Response:** We have clarified in the **"Strengths and Limitations"** section that temporal validation is not a substitute for external validation. To address the handling of concept drift, we have added a new analysis (**Table 6**) and a corresponding discussion that identifies the shift in patient characteristics (a decrease in third-degree burns) as the likely driver of the observed performance shift.

**Reviewer #5**

**Comment 1: Data Leakage in Temporal Split.**

- **Response:** We have rewritten the **"Temporal Validation"** section in the Methods to explicitly state that the cohorts were divided chronologically and were **"non-overlapping,"** ensuring that each unique patient belonged to only one group.

**Comment 2: SHAP Analysis.**

- **Response:** As noted in our response to Reviewer #3, we were unable to perform SHAP analysis due to technical limitations. We have addressed this in the **"Strengths and Limitations"** section and have provided a clear alternative visualization (Figure 3) and a deeper discussion of the feature importance results.

We thank the editor and reviewers again for their invaluable guidance. We are confident that the revised manuscript is now a much stronger, more accurate, and more impactful contribution to the field, and we hope it is now suitable for publication in PLOS ONE.

Sincerely,

Asghar Ehteshami (on behalf of all authors)
