# Supplementary material for: From data to decisions: Predicting inpatient burn mortality with advanced classification models
Source: PLoS One. 2026 Jan 2;21(1):e0338564. doi: 10.1371/journal.pone.0338564 (PMC12758681; doi:10.1371/journal.pone.0338564)
Supplement: Attachment — Submitted filename: Response to Reviewers-14040829.docx [file pone.0338564.s013.docx]

**Cover Letter / Response to Reviewers**

**Date:** 20-11-2025 **To:** Dr. Amirreza Khalaji, Academic Editor, PLOS ONE **Manuscript ID:** PONE-D-25-02698R2 **Title:** From Data to Decisions: Predicting Inpatient Burn Mortality with Advanced Classification Models

**Dear Dr. Khalaji and distinguished Reviewers,**

Thank you for providing us with the opportunity to revise our manuscript. We are grateful for the thoughtful and detailed feedback from the review team, which has significantly enhanced the rigor and transparency of our work. We are pleased to note the positive consensus among the majority of reviewers regarding the manuscript’s suitability for publication.

In this revision, we have focused on addressing the methodological and reporting standards raised by Reviewer #6, as well as the visualization requests from Reviewer #8. Specifically, we have:

1. Included a detailed schematic diagram of the validation pipeline (**Fig 4**) to clarify data handling procedures.
2. Added a visual comparison of model performance (**Fig 5**) to highlight the impact of concept drift.
3. Calculated and reported **95% Confidence Intervals** for the temporal validation metrics in **Table 5**.
4. Completed the **TRIPOD Checklist** (provided as **S1 Checklist**) to ensure standardized reporting.
5. Refined the **Data Availability Statement** and standardized references as requested.

Below, we provide a point-by-point response to the specific comments raised in this round.

**Response to Reviewer #6**

**Comment 1:** *Model comparisons and statistical testing (limitations of paired t-tests).* **Response:** We appreciate the reviewer's statistical insight. We agree that cross-validation folds are not strictly independent, which can inflate Type I error rates in paired t-tests. To address this concern:

- We have shifted the statistical weight of our evidence towards the **Temporal Validation** results, which are based on an independent, chronologically distinct test cohort (n=216).
- Instead of relying solely on CV-based p-values, we have calculated and reported **95% Confidence Intervals (CIs)** for the temporal validation metrics (Accuracy, Sensitivity, Specificity, Precision) using the **Wilson Score Interval** method. These are now explicitly presented in **Table 5** (e.g., Sensitivity: 68.29% [95% CI: 53.0% – 80.4%]).
- We have added a statement in the **"Strengths and Limitations"** section explicitly acknowledging the limitations of fold-based statistical comparisons.

**Comment 2:** *Temporal validation protocol and leakage safeguards (Schematic diagram).* **Response:** We have added a comprehensive schematic diagram (**Fig 4**) to visually detail the study pipeline.

- **Pipeline Clarification:** As illustrated in Fig 4, all feature selection, one-hot encoding rules, and hyperparameter tuning were derived strictly from the **Training Cohort** (first 9 months). The final classification models (GBT/RF) were frozen and applied to the test cohort without retraining.
- **Imputation Strategy:** Regarding the GLM imputation, we identified a significant temporal concept drift in the dataset (e.g., statistical changes in burn severity). Furthermore, due to technical constraints in our specific analytical environment preventing the direct serialization and transfer of the trained GLM object, we opted to perform **Independent GLM Imputation** on the temporal test cohort. This methodological choice ensures that missing values in the test set are imputed based on the concurrent data structure (respecting the temporal shift) rather than enforcing the distributional assumptions of the past training set. This rationale has been explicitly added to the "Strengths and Limitations" section.

**Comment 3 & 6:** *Uncertainty, Calibration, and Performance Reporting.* **Response:**

- **Uncertainty:** As noted above, **95% CIs** for key performance metrics on the temporal test set have been added to **Table 5**.
- **Calibration:** We previously reported the **Brier Score** (0.060 for GBT), which indicates good calibration. Regarding the request for calibration plots or rank-based CIs (e.g., for AUC): Due to data storage constraints in our deployed environment where raw probability logs for the historical test set were not retained, we could not perform post-hoc bootstrapping or generate calibration curves. We have reported AUC and Brier Score as point estimates and have transparently noted this limitation in the text.

**Comment 4:** *Decision threshold.* **Response:** We have clarified in the **Methods** section (under *Model Performance Evaluation*) that the standard decision threshold of **0.5** was applied for all classification models to convert probabilities into class labels.

**Comment 5:** *Tuning objective aligned to clinical goals (Suggestion to use AUC instead of Accuracy).* **Response:** We appreciate this valid methodological point. Ideally, for mortality prediction, optimizing for AUC or Recall is preferable. However, we respectfully submit that in our specific dataset, the class imbalance was moderate (22.1% mortality rate) rather than extreme. Empirically, optimizing for **Accuracy** did not result in a biased model favoring the majority class; our final GBT model achieved a high **AUC of 0.966** and a robust **Sensitivity (Recall) of 78.1%** in the development phase. This demonstrates that in this specific feature space, Accuracy was strongly correlated with discriminative power. Regarding the reviewer’s question on class weights: **No class weights** were applied during training, as the model demonstrated satisfactory sensitivity without artificial re-balancing. We have maintained the current optimization due to technical constraints preventing a full re-execution of the nested grid search, but we believe the final performance metrics validate the utility of the chosen models.

**Comment 7, 8 & 9:** *TRIPOD, Data Availability, and Formatting.* **Response:**

- **TRIPOD:** A completed **TRIPOD Checklist** has been uploaded as **S1 Checklist**, and a statement of adherence has been added to the Methods section.
- **Data Availability:** The Data Availability Statement in the manuscript has been updated to align exactly with the metadata in the submission system, providing the official contact email for the Ethics Committee ([resrec@mui.ac.ir](mailto:resrec@mui.ac.ir)). Additionally, regarding Code Availability, please note that the Zenodo DOI mentioned in the manuscript serves as a permanent archive and is currently in 'Restricted Access' pending publication. Therefore, for the purpose of peer review, the RapidMiner model file has been directly uploaded to the submission system as a 'Supporting Information' file.
- **Formatting:** Decimal marks have been corrected (standardized to dots), and references have been formatted with DOIs where available.

**Response to Reviewer #7**

**Comment:** *Recommend adding TreeSHAP and Multiple Imputation (MI).* **Response:** We thank the reviewer for these advanced suggestions.

- **SHAP:** Unfortunately, the specific version of the RapidMiner platform available for this study does not currently support the Python integration required for TreeSHAP libraries. To address interpretability, we have relied on the model-specific **Feature Importance** plot (**Fig 3**), which robustly identifies key predictors.
- **Multiple Imputation:** While MI is a powerful technique, our sensitivity analysis (reported in **Table 7**) demonstrated that the model's performance was robust to the choice of imputation method (GLM vs. Mean). Given the resource constraints, we believe the current GLM approach is sufficient to demonstrate the model's validity.

**Response to Reviewer #8**

**Comment 1:** *Visualize results with bar plots.* **Response:** We have added **Fig 5**, a bar chart that visually compares the performance of the **Main Model** (derived from 10-fold CV) against the **Temporal Validation** results. This figure clearly highlights the stability of Accuracy and Specificity alongside the notable drop in Sensitivity due to concept drift.

**Comment 2:** *Simplify the features table.* **Response:** We agree that the detailed descriptive statistics were lengthy for the main text. We have moved the full detailed table to the Supporting Information as **S1 Table** and replaced it in the main text with a concise **Summary Table (Table 2)** that lists variable categories and data handling methods.

**Conclusion** We believe that these revisions, particularly the enhanced transparency regarding the validation pipeline and the robust reporting of uncertainty, have significantly strengthened the manuscript. We thank the editor and reviewers for guiding us toward this improved version.

Sincerely, **Asghar Ehteshami** (On behalf of all authors)
